# Evidence Transcranial Direct Current Stimulation Can Improve Saccadic Eye Movement Control in Older Adults

**DOI:** 10.3390/vision2040042

**Published:** 2018-12-03

**Authors:** Po Ling Chen, Andreas Stenling, Liana Machado

**Affiliations:** 1 Department of Psychology and Brain Health Research Centre, University of Otago, Dunedin 9054, New Zealand; 2 Brain Research New Zealand, Auckland 1142, New Zealand; 3 Department of Psychology, Umeå University, 901 87 Umeå, Sweden; 4 Department of Psychology, University of Gothenburg, SE405 30 Gothenburg, Sweden

**Keywords:** electrical brain stimulation, oculomotor control, saccade, antisaccade, online tDCS

## Abstract

**Objectives:** Ageing is associated with declines in voluntary eye movement control, which negatively impact the performance of daily activities. Therapies treating saccadic eye movement control deficits are currently lacking. To address the need for an effective therapy to treat age-related deficits in saccadic eye movement control, the current study investigated whether saccadic behaviour in older adults can be improved by anodal transcranial direct current stimulation (tDCS) over the dorsolateral prefrontal cortex using a montage that has been proven to be effective at improving nonoculomotor control functions. **Method:** The tDCS protocol entailed a 5 cm × 7 cm anodal electrode and an encephalic cathodal reference electrode positioned over the contralateral supraorbital area. In two experiments, healthy older men completed one active (1.5 mA current for 10 min) and one sham stimulation session, with the session order counterbalanced across participants, and eye movement testing following stimulation. In the first experiment, participants rested during the tDCS (offline), whereas in the follow-up experiment, participants engaged in antisaccades during the tDCS (online). **Results:** Analyses revealed improvements in saccadic performance following active anodal tDCS relative to sham stimulation in the online experiment, but not in the offline experiment, which was presumably due to the activation of the relevant networks during tDCS promoting more targeted effects. **Discussion:** These outcomes converge with findings pertaining to nonoculomotor cognitive functions, and provide evidence that tDCS can improve saccadic eye movement control in older adults.

## 1. Introduction

Ageing is associated with a decline in saccadic eye movement control [1,2]. Deficits are particularly notable when the eye movement requires a high level of voluntary control, such as when the inhibition of eye movement reflexes is needed [3,4,5]. Such declines in strategic control over saccadic eye movements have negative implications for the performance of time-sensitive tasks that rely on effective visual orienting, such as driving a motorised vehicle [6,7] or navigating a busy intersection [8]. To date, there is a dearth of studies investigating methods to counteract oculomotor deficits in older adults, despite reports of age-related deficits being larger for voluntary control over eye movements relative to nonoculomotor cognitive domains (e.g., working memory [9]).

Transcranial direct current stimulation (tDCS) has been put forward as a technique that has the potential to mitigate cognitive deficits, including oculomotor control difficulties in ageing brains [2]. Kanai et al. [10] provided initial evidence in support of this possibility in healthy young adults. Using a small anodal electrode (3 cm × 3 cm) that delivered one mA of electrical current over the left or right frontal eye field (FEF) for 10 min, they found evidence of improved saccadic eye movement behaviour contralateral to the stimulated hemisphere. In their study, the FEF was localised using magnetic resonance imaging (MRI), and the extracephalic reference electrode was placed over the shoulder (i.e., deltoid muscle) ipsilateral to the anodal electrode. Building on the young adult findings reported in Kanai et al. [10], Chen and Machado [11] modified the protocol to make it more clinically practical (by removing the use of expensive tools and time-consuming procedures), and trialled it in healthy young and older adults. However, the results showed no benefits of anodal tDCS on saccadic eye movement behaviour in either age group.

The null results in the older age group in Chen and Machado [11] came as a particular surprise in light of suggestions that tDCS can confer greater cognitive benefits in older adults [12] because they have far more room for improvement due to age-related cognitive decline. However, the failure to improve older adults’ oculomotor behaviour using a small anodal electrode could make sense given that ageing brains have been found to rely on widespread perfusion in prefrontal regions [13], especially when processing higher-level cognitive tasks [14]. Thus, stimulating more focally using a small anodal electrode may not induce the required physiological changes in prefrontal regions to influence saccadic eye movement control in older adults.

To address this, the current study trialled a protocol designed to suit not only clinical settings, but also the physiological conditions that are typical in older adult brains. In order to target a more widespread region, we utilised a large (5 cm × 7 cm) anodal electrode placed over the prefrontal cortex. Jones et al. [15] demonstrated using current modelling that this size of electrode placed over the prefrontal cortex—delivering 1.5 mA of anodal direct current for 10 min and paired with a reference electrode of the same size placed over the contralateral cheek—allowed the current to flow within prefrontal regions in older adults. Current modelling also indicated that this protocol supplied current not only to the area underlying the anodal electrode, but also to surrounding areas within the prefrontal regions. In this study, we placed the encephalic reference electrode over the contralateral supraorbital area (i.e., forehead) instead of the cheek to avoid current passing through the eye, just in case this might affect eye movements. This positioning of the reference electrode should also allow the current to flow through prefrontal regions on its path from the anodal to the reference electrode, with a prefrontal electric field concentration [16]. Moreover, this tDCS montage has been shown in a number of past studies to improve nonoculomotor cognitive functions (as summarised in Prehn & Flöel [17]; Teixeira-Santos et al. [18]).

With respect to simplifying the tDCS protocol to be more logistically practical in non-research settings, as per Chen and Machado [11], rather than using MRI to determine the positioning of the anodal electrode, the current study used simple measurements based on the 10–20 electroencephalography (EEG) system [19], which is both quicker and less expensive than MRI. By using a larger sized electrode centred over dorsolateral prefrontal cortex (DLPFC), in contrast to the smaller sized 3 cm × 3 cm electrode that was used previously [10,11], we aimed not only to influence the overall network, supporting effective control over saccadic eye movements in older adults [2], as discussed earlier, but also to obviate the need for precision measurement. In addition, in light of recent evidence indicating that engaging in the relevant behaviour during the tDCS can enhance the benefits gained [20,21,22,23,24], in the current study, we included a follow-up experiment during which participants engaged in eye movements during the period of tDCS (online), as opposed to resting (offline). The rationale for online tDCS being superior to offline tDCS is that activation of the relevant neural networks during tDCS can help promote more targeted effects [21,25,26]. We predicted that, relative to sham tDCS, active DLPFC stimulation would improve saccadic eye movement behaviour during the post-stimulation period, which showed evidence of performance improvements in Kanai, Muggleton, and Walsh [10] (see Figure 4 in reference). Moreover, we predicted that engaging in saccadic eye movements during active tDCS would enhance the post-stimulation benefits.

## 2. Methods

This study was approved by the University of Otago Human Ethics Committee (Dunedin, New Zealand) (H13/123). All participants gave informed consent prior to participation, and all methods were performed in accordance with the relevant guidelines and regulations and the Declaration of Helsinki.

### 2.1. Participants

The final sample included 16 males (age range = 65–71 years, *M* = 68.4, *SD* = 1.7; education range = 10–20 years, *M* = 14.6, *SD* = 3.4) who completed the first experiment (offline) and 10 new males (age range = 65–74 years, *M* = 68.4, *SD* = 2.5; education range = 10–23 years, *M* = 15.2, *SD* = 3.8) who completed the follow-up experiment (online). One additional participant completed the offline experiment, and two additional participants completed the online experiment, but were excluded due to an inability to perform the antisaccade task; these three participants will not be discussed further. All were from the Dunedin community in New Zealand, and were reimbursed NZ$15 per session. According to the Measurement of Handedness [27], all participants in both experiments were right-handed except for one (ambidextrous) in the offline experiment. All participants reported having normal or corrected vision; no pacemaker or implanted electronic device; no history of, and not currently taking any medications for neurological or psychiatric problems (except for one participant in the offline experiment who was on depression medication); no chronic skin conditions or current skin irritation in the areas of stimulation; and abstained from recreational drugs and more than three units of alcohol during the 24 h prior to their testing session. All participants completed the Center for Epidemiologic Studies Depression Scale (CES-D) [28], which has a maximum score of 60: in the offline experiment, 13 participants scored below 16, indicating that they had no clinical symptoms of depression, and three participants scored between 16–20, indicating subthreshold depression symptoms; in the online experiment, six participants scored below 16, and four participants scored between 16–21. All participants were screened for dementia using the Mini-Mental State Examination [29]; all scored at least 25 out of 30, which suggests that none of the participants were demented.

### 2.2. Design

The offline and online experiments were identical except that in the online experiment, participants engaged in antisaccades during the tDCS (as detailed in the next section). Both experiments employed a participant-blinded, sham-controlled randomised crossover design. Half of the participants in each experiment were randomly assigned to left hemisphere stimulation and half were assigned to right. All participants completed two sessions, one involving active stimulation and one involving sham stimulation (always with the anodal electrode positioned over the DLPFC of the assigned hemisphere), with the order of the sessions counterbalanced across the participants in each experiment, and the two sessions separated by a minimum of seven days. Each session lasted about one hour.

### 2.3. tDCS Protocol

**Figure 1:**
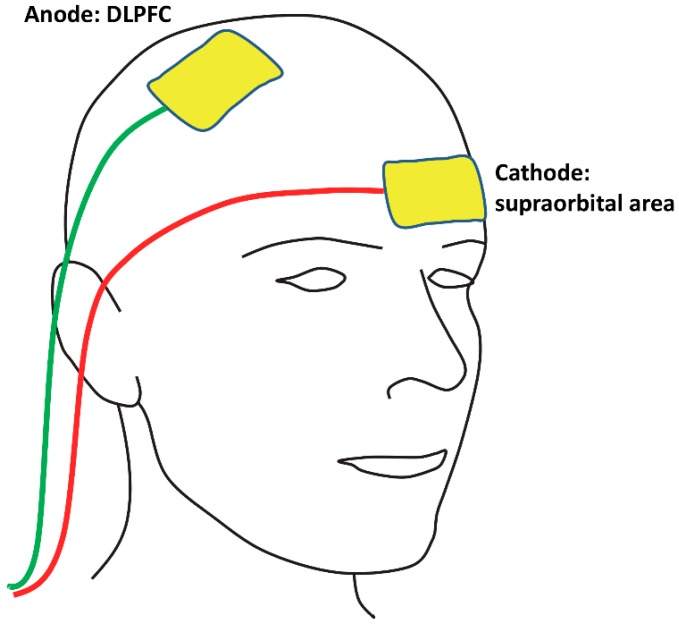
An illustration of the electrode montage. In this example, the anodal electrode is over the right dorsolateral prefrontal cortex (DLPFC) and the reference electrode (i.e., cathode) is over the contralateral supraorbital area, but note that for half of the participants in each experiment, the sides of the electrodes were reversed (anode over the left DLPFC and cathode over the right supraorbital area). During active stimulation, 1.5 mA of current was delivered for 10 min, and during sham stimulation, the device was turned off 30 s after the start of stimulation.

The 10–20 system for EEG [19] was used to determine the placement of the anodal electrode over the assigned hemisphere. The position of DLPFC was defined relative to the vertex: five centimetres anterior and 20% lateral [30]. This site was used for the anodal electrode during both active and sham stimulation, and the reference electrode (cathode) was positioned on the supraorbital area (i.e., forehead above the eye) contralateral to the anodal stimulation site (as shown in Figure 1). Prior to proceeding, inspection of all sites of stimulation confirmed that there were no lesions or signs of skin irritation.

A constant current nine-volt battery-driven device (ActivaDose II) delivered 1.5 mA of direct current through carbon rubber electrodes placed in sponge pockets soaked in saline solution. Both the anodal and reference electrodes were 5 cm × 7 cm in size and delivered a current density of 0.043 mA/cm^2^. The intensity of the current slowly ramped up to 1.5 mA over the initial 15 s of stimulation. During active stimulation, current was delivered for 10 min, and during sham stimulation, the device was turned off 30 s after the start of the stimulation. In the online experiment only, after the first five minutes of each stimulation condition, participants completed 40 antisaccade trials (data not recorded), which took approximately two minutes. At the end of each stimulation period, participants completed a questionnaire designed to monitor adverse effects and participants’ subjective experience of tDCS stimulation. No participants reported adverse effects or, when questioned at the end of their final session, that they were able to distinguish between the two stimulation conditions (active versus sham).

### 2.4. Eye Tracking Protocol

**Figure 2:**
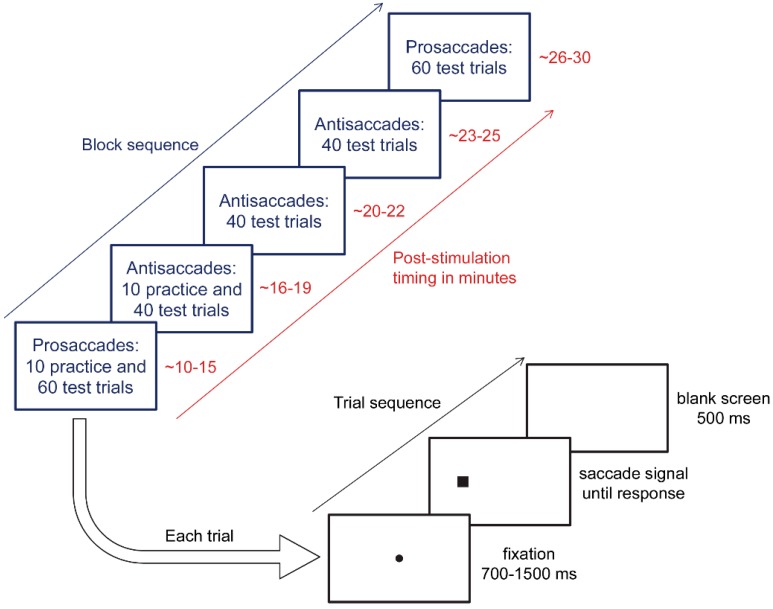
Eye tracking protocol adapted from Antoniades et al. [31].

The eye movement paradigms were adapted from Antoniades et al. [31], and were used previously in Chen and Machado [11]. Eye movement testing commenced 10 min post-stimulation in order to target the post-stimulation time period that showed anodal tDCS benefits in Kanai, Muggleton, and Walsh [10]. Participants completed five blocks of eye movements in this sequential order: one prosaccade block, three antisaccade blocks, and then a second prosaccade block (see Figure 2). Each prosaccade test block had 60 trials, and each antisaccade test block had 40 trials, and participants were given 10 practice trials at the beginning of the first block of each saccade type. Practice trials were repeated if the participant requested or did not appear to understand the instructions. Between blocks, participants were provided with a one-minute break. Between blocks of different types, the experimenter instructed participants in how to respond. Participants wore a head-mounted eye tracker (Model 310, Applied Science Laboratories, Bedford, MA, USA) and sat 57 cm away from a computer screen in a dimly lit room, with distance maintained via a chinrest. The experimenter calibrated the eye-tracking system before each block. Stimuli were presented on a white background via MATLAB (The MathWorks, Natick, MA, USA) and The Psychophysics Toolbox [32,33].

Figure 2 depicts the sequence of each eye movement trial. First, a black fixation dot extending 0.3° of visual angle appeared at the centre of the screen. After a variable interval (700 ms, 900 ms, 1100 ms, 1300 ms, or 1500 ms), the fixation dot disappeared, and at the same time, a black square subtending 1° appeared 8.5° to the left or right of centre (measured to the centre of the square). Participants were instructed to respond to the appearance of the square as quickly as they could without compromising accuracy by looking at it during prosaccade blocks, and by looking in the opposite direction during antisaccade blocks. During practice trials only, a 900-Hz error tone sounded for 300 ms if the participants made no response, responded in the wrong direction, or responded in less than 50 ms or more than 1000 ms after saccade signal onset. The screen went blank for 500 ms between trials. Saccade signal position (left or right) and fixation duration (700 ms, 900 ms, 1100 ms, 1300 ms, or 1500 ms) were randomly selected for each trial, with the constraint that each combination of conditions was equally likely to occur across the test trials in each block.

The horizontal position of the right eye was sampled at 1100 Hz. When the right eye exceeded the horizontal velocity of 50°/s with at least 1° amplitude, the movement was defined as a saccade. The program then recorded the latency of saccade onset (by backtracking until the velocity dropped below 10°/s) and the direction of movement. During the trials, the experimenter manually rejected responses contaminated by blinking or other disruptions. In addition, trials were excluded from analysis if the eye position at the time of the saccade signal onset deviated from the centre by more than 3°, or if the latency was shorter than 50 ms or longer than 1000 ms.

### 2.5. Statistical Analyses

For each participant, the measured variables of interest were median reaction times (RTs) for correct prosaccades and antisaccades, and the percentage of reflexive errors during antisaccade blocks (i.e., erroneous stimulus-directed saccades) as a function of stimulation condition (active or sham) and saccade direction (ipsilateral or contralateral to the anodal electrode). The Shapiro–Wilk test was used to determine the normality of each dataset. When assumptions of parametric tests were violated, non-parametric tests were performed to confirm the parametric results. In cases where sphericity was violated (*p* < 0.05), a Greenhouse–Geisser correction was applied when Epsilon ranged from 0.70 to 0.90; otherwise, a multivariate test (Pillai’s trace) was applied. Each measured variable of interest was first subjected to a repeated-measures analysis of variance (ANOVA), followed by a paired-samples *t* test that compared the active and sham stimulation conditions (with effect sizes indicated by Cohen’s *d_z_*). In line with recommendations, we used one-tailed *t* tests, because our hypotheses involved directional predictions [34], and one-tailed tests are more powerful for directional hypotheses compared to two-tailed tests [35]. These analyses were conducted using the IBM Statistical Package for the Social Sciences (SPSS) 23, except for Cohen’s *d_z_*, which was calculated using the formulas outlined in Lakens [36]. The alpha level was set to 0.05.

For the *t* tests, we also calculated Bayes factors (BF_10_) to examine the likelihood of the data under the null (H_0_) and alternative (H_1_) hypothesis, respectively [37]. Bayes factors were calculated using Bayesian-dependent sample *t* tests [38] in JASP version 0.8.4.0 (JASP Team, 2017) using a symmetrical Cauchy prior, centred on zero (*r* = 0.707, which is the default in JASP). With regard to interpretation, a BF_10_ larger than three indicates evidence for H_1_ (three to 10 = moderate evidence, 10–30 = strong evidence, >30 = very strong evidence), whereas a BF_10_ smaller than 0.33 indicates evidence for H0 (0.10–0.33 = moderate evidence, 0.03–0.10 = strong evidence, <0.03 = very strong evidence). A BF_10_ between 0.33–3 is considered anecdotal (i.e., inconclusive [39]). The sample size for the initial experiment (offline) was based on the study in young adults [10], which included 16 participants and showed post-stimulation improvements in oculomotor control, as this is the only study to date reporting saccadic eye movement control benefits from tDCS. For the follow-up study, resources prevented us from meeting this sample size.

## 3. Results

**Figure 3:**
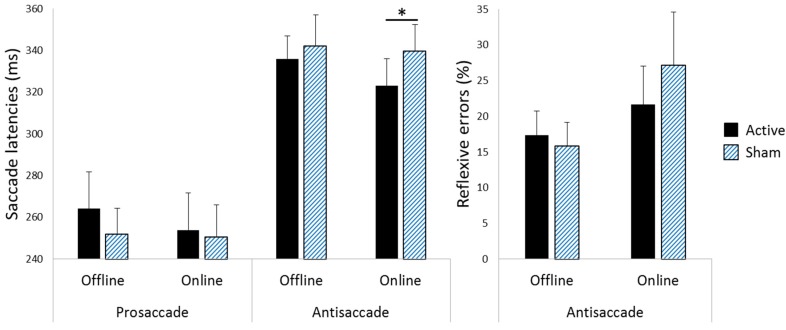
Performance following active versus sham stimulation in the offline and online experiments. Antisaccade latencies were significantly reduced following active compared to sham stimulation in the online, but not the offline, experiment. Bars indicate standard errors. * *p* = 0.018, BF_10_ = 4.493.

Table 1 summarises the mean of the median correct prosaccade and antisaccade response latencies and reflexive error rates during antisaccade blocks for each saccade direction following each stimulation condition in each experiment. Figure 3 illustrates the differences between the offline and the online experiments in antisaccade latencies following active versus sham stimulation. Initial analyses showed that in both experiments, neither the stimulated hemisphere nor saccade block interacted with the stimulation conditions or saccade direction (*p* > 0.05 in all cases).

### 3.1. Saccade Latencies

**Offline Experiment.** A repeated-measures ANOVA with stimulation condition, saccade type, and saccade direction as factors confirmed the expected main effect of saccade type, *F*(1, 15) = 80.712, *p* < 0.001, *r* = 0.918, reflecting longer latencies during antisaccades than prosaccades. However, there was no significant main effect of stimulation condition, *F*(1, 15) = 0.414, *p* = 0.530, *r* = 0.164. Of particular relevance, stimulation condition neither interacted with saccade type, *F*(1, 15) = 0.837, *p* = 0.375, *r* = 0.230, nor with saccade direction, *F*(1, 15) = 0.048, *p* = 0.829, *r* = 0.055. Furthermore, there was no three-way interaction between stimulation condition, saccade type, and saccade direction, *F*(1, 15) = 0.029, *p* = 0.867, *r* = 0.045. Together, these results indicate that prosaccade and antisaccade latencies were not differentially affected by active versus sham stimulation. No other main effects or interactions were statistically significant (all *p*s >0.200). Paired-samples *t* tests confirmed no improvements following active versus sham stimulation in the latencies of prosaccades, *t*(15) = 0.992, *p* = 0.169 [one-tailed], Cohen’s *d_z_* = 0.248, BF_10_ = 0.143, or antisaccades, *t*(15) = −0.656, *p* = 0.261 [one-tailed], Cohen’s *d_z_* = −0.164, BF_10_ = 0.449.

**Online Experiment.** A repeated-measures ANOVA with stimulation condition, saccade type, and saccade direction as factors confirmed the expected main effect of saccade type, *F*(1, 9) = 31.350, *p* < 0.001, *r* = 0.881, reflecting longer latencies during antisaccades than prosaccades. The main effect of stimulation condition was not statistically significant, *F*(1, 9) = 1.476, *p* = 0.255, *r* = 0.375; however, stimulation condition interacted with saccade type, *F*(1, 9) = 9.300, *p* = 0.014, *r* = 0.713. Stimulation condition did not interact with saccade direction, *F*(1, 9) = 0.390, *p* = 0.548, *r* = 0.205; there was no three-way interaction between stimulation condition, saccade type, and saccade direction, *F*(1, 9) = 0.047, *p* = 0.832, *r* = 0.071, and no other main effects or interactions were statistically significant (all *p*s >0.100). Thus, to follow up the interaction between stimulation condition and saccade type, we collapsed across saccade direction and ran paired-samples *t* tests comparing active to sham for each type of saccade, which revealed that although prosaccade latencies did not differ following active versus sham stimulation, *t*(9) = 0.486, *p* = 0.320 [one-tailed], Cohen’s *d_z_* = 0.154, BF_10_ = 0.143, antisaccade latencies were significantly reduced following active relative to sham stimulation, *t*(9) = −2.477, *p* = 0.018 [one-tailed], Cohen’s *d_z_* = −0.783, BF_10_ = 4.493.

### 3.2. Reflexive Error Rates during Antisaccade Blocks

**Offline Experiment.** A repeated-measures ANOVA with stimulation condition and saccade direction as factors showed neither main effects of stimulation condition, *F*(1, 15) = 1.186, *p* = 0.293, *r* = 0.270, and saccade direction,* F*(1, 15) = 0.029, *p* = 0.867, *r* = 0.045, nor an interaction between stimulation condition and saccade direction, *F*(1, 15) = 0.679, *p* = 0.423, *r* = 0.207, which indicates that ipsilateral versus contralateral reflexive error rates were not differentially influenced by active versus sham stimulation. Thus, we collapsed the data across saccade direction, and then assessed the stimulation condition via a paired-samples *t* test, which showed no differences in reflexive error rates following active versus sham stimulation, *t*(15) = 1.089, *p* = 0.147 [one-tailed], Cohen’s *d_z_* = 0.272, BF_10_ = 0.137.

**Online Experiment.** A repeated-measures ANOVA with the stimulation condition and saccade direction as factors showed neither the main effects of stimulation condition, *F*(1, 9) = 3.338, *p* = 0.101, *r* = 0.521, and saccade direction,* F*(1, 9) = 1.949, *p* = 0.196, *r* = 0.422, nor an interaction between stimulation condition and saccade direction, *F*(1, 9) = 0.865, *p* = 0.377, *r* = 0.297, which indicates that ipsilateral versus contralateral reflexive error rates were not differentially influenced by active versus sham stimulation. Thus, we collapsed the data across saccade direction, and then assessed the stimulation condition via a paired-samples *t* test, which showed a trend for reduced reflexive errors following active relative to sham stimulation. However, the difference was not statistically significant, *t*(9) = −1.827, *p* = 0.051 [one-tailed], Cohen’s *d_z_* = −0.578, BF_10_ = 1.960.

## 4. Discussion

The current study investigated whether saccadic eye movement control can be improved using a tDCS protocol tailored to suit older adults (i.e., brains with more distributed neural networks) and non-research settings (by using a quick and inexpensive electrode positioning procedure). Saccadic eye movement control was assessed during the post-stimulation period, and showed the most robust evidence of anodal tDCS benefits in young adults [10]. The results showed improvements in oculomotor control following online tDCS, for which participants engaged in antisaccades during the stimulation. The post-stimulation improvements were restricted to antisaccade performance; these were reflected in faster latencies for correct antisaccades and a trend for fewer erroneous reflexive saccades [note that this overall pattern is consistent with findings in young adults using a small anodal electrode [10], see Figures 3 and 4]. In contrast to the online experiment, the offline experiment revealed no significant improvements in saccadic eye movement control following active versus sham stimulation. Thus, taken together, our findings suggest that engaging in saccadic eye movement control during tDCS may offer more therapeutic advantage than offline protocols.

The evidence of benefits following online, but not offline, tDCS converges with findings related to nonoculomotor control functions, indicating that tDCS paired with task engagement yields greater benefits [20,21,22,23]. The performance improvements reported here following online active versus sham stimulation were restricted to the task performed during the tDCS (i.e., antisaccade, but not prosaccade, performance improved), which could indicate that improvements hinged on participants engaging in the task during the tDCS. If so, the performance of prosaccades during the tDCS would presumably have led to subsequent prosaccade improvements, which is consistent with findings in young adults [10]. However, given that antisaccades are more cognitively challenging than reflexive prosaccades, and they also show greater decline with adult ageing [2,4], it could be the case that the observed performance improvements hinged on the nature of the task rather than on the particular task being performed during the tDCS. This conjecture fits with DLPFC playing a stronger role in the performance of antisaccades than reflexive prosaccades, as evidenced by neuroimaging and human lesion studies [2]. Moreover, our finding of improved antisaccade but not prosaccade performance following DLPFC electrical brain stimulation fits with past transcranial magnetic stimulation (TMS) studies that reported that TMS over the prefrontal cortex disrupted voluntary eye movement control, but not reflexive prosaccades [40]. Although the trend for a reduction in reflexive errors during the antisaccade task did not reach statistical significance, the medium-sized effect is in line with past reports that suggested that damage to the frontal cortex can cause increases in erroneous reflexive errors during antisaccade tasks (e.g., Machado & Rafal [41]). Given that the trend for fewer reflexive errors occurred in combination with faster correct antisaccade latencies, it may be that the tDCS in the current study facilitated the attenuation of salience representations, which is consistent with past evidence of salience modulation by TMS (e.g., Lane et al. [42]).

Excitement about this evidence that anodal tDCS can improve saccadic eye movement control in older adults must be tempered by the limitations of this study. First and foremost is the small sample size. Although the Bayes factor indicates that the data indicating that improved antisaccade latencies are over four times more likely under H_1_ than under H_0_, the effect size of the observed performance benefit was fairly large [43], and a post hoc power calculation showed that 74% power was achieved with the final sample size of 10 participants for the online experiment. Nonetheless, it is clearly necessary to replicate our findings in a larger cohort. Second, none of the participants were left-handed or female; thus, additional research will be needed to determine whether the current findings can be generalised to these populations. Third, the minimum washout period was only seven days; thus, carryover effects may have watered down differences between the active versus sham stimulation conditions, given that some studies have detected tDCS aftereffects beyond a week (reviewed in Hurley & Machado [24]). Fourth, although the current study adopted an internationally standardised antisaccade protocol [31], which may be viewed as a strength, this does not preclude the possibility that the antisaccade benefits detected are specific to the eye movement protocol that was used. However, the similarities with Kanai, Muggleton, and Walsh [10] in the pattern of antisaccade effects suggest that the findings may be robust to the specific eye movement protocol. Fifth, over 10% of the participants who were recruited had to be excluded due to an inability to perform the antisaccade task; thus, efforts may be needed to modify the antisaccade protocol to suit people who experience difficulties. Sixth, post-tDCS oculomotor testing ended approximately 30 min after active tDCS terminated; thus, the current study cannot inform about potential longer-term benefits, which will be important to establish through future research. Seventh, the timing of the oculomotor assessments was centred around the most robust anodal tDCS effects in young adults [10]; however, recent research indicates that the effects of anodal tDCS may be delayed in older compared to young adults, as peak motor excitability effects occurred 30 min post-stimulation in older adults, but immediately post-stimulation in young adults [44]. Thus, future studies in older adults may opt to probe eye movement control at longer post-stimulation intervals. Additionally, given that electric field strength negatively correlates with age [45], future studies may consider increasing the stimulation duration and current intensity, as these variables have been shown to magnify the aftereffects of tDCS [46], which may be particularly needed in older adults.

In conclusion, this study is the first to report evidence that anodal tDCS may provide an effective therapy to ameliorate saccadic eye movement control deficits in older adults. The results indicate improvements in oculomotor control following online active relative to sham tDCS. That improvements were restricted to the online protocol suggests that the endogenous activation of the relevant brain networks during the stimulation may have promoted more targeted tDCS effects, resulting in a therapeutic advantage that converges with findings pertaining to nonoculomotor control functions (e.g., Stagg et al. [20]; Mancuso et al. [21]; Martin et al. [22]; Oldrati et al. [23]). Thus, future research aiming to enhance the benefits from tDCS should consider including online engagement in the protocol. These initial indications of a protocol that is effective at improving saccadic eye movement control in healthy older adults bode well with respect to future prospects of developing protocols that are effective in neurological patients suffering from oculomotor control difficulties, for example in relation to Parkinson’s disease [47] or frontal lobe stroke [41,48].

## Figures and Tables

**Table 1:** Saccade latencies (in milliseconds) and reflexive errors (in percentage) following active versus sham stimulation in each experiment.

	Offline Experiment (*n* = 16)				Online Experiment (*n* = 10)			
	Active		Sham		Active		Sham	
	Ipsi	Contra	Ipsi	Contra	Ipsi	Contra	Ipsi	Contra
Prosaccade RTs (ms)	258 (73)	270 (79)	246 (49)	258 (54)	260 (59)	248 (56)	256 (59)	246 (40)
Antisaccade RTs (ms)	335 (46)	337 (45)	340 (53)	344 (71)	329 (53)	317 (33)	342 (53)	337 (35)
Reflexive Errors (%)	18.3 (14.8)	16.4 (16.3)	15.5 (13.4)	16.1 (18.1)	20.2 (19.7)	23.0 (15.8)	24.0 (25.0)	30.3 (23.8)

Note: Ipsi = saccade directed ipsilateral to the anodal electrode; Contra = saccade directed contralateral to the anodal electrode; RTs = reaction times. Reflexive errors refer to antisaccade blocks. Standard deviations are listed in parentheses.
